# Identification of adeno-associated virus variants for gene transfer into human neural cell types by parallel capsid screening

**DOI:** 10.1038/s41598-022-12404-0

**Published:** 2022-05-19

**Authors:** Lea Jessica Flitsch, Kathleen Börner, Christian Stüllein, Simon Ziegler, Vera Sonntag-Buck, Ellen Wiedtke, Vesselina Semkova, Si Wah Christina Au Yeung, Julia Schlee, Mohamad Hajo, Mona Mathews, Beatrice Stefanie Ludwig, Susanne Kossatz, Horst Kessler, Dirk Grimm, Oliver Brüstle

**Affiliations:** 1 Institute of Reconstructive Neurobiology, University of Bonn Medical Faculty and University Hospital Bonn, Venusberg-Campus 1, Building 76, 53127 Bonn, Germany; 2 Center for Infectious Diseases, Virology, Medical Faculty, Heidelberg University, Im Neuenheimer Feld 344, 69120 Heidelberg, Germany; 3 BioQuant, Heidelberg University, Im Neuenheimer Feld 267, 69120 Heidelberg, Germany; 4 German Center for Infection Research (DZIF), partner site Heidelberg, 69120 Heidelberg, Germany; 5 CLADIAC GmbH, Kurfürsten-Anlage 52-58, 69115 Heidelberg, Germany; 6 Stüllein Software Engineering (SSE), Friedrich-Hartung-Str. 16, 64560 Riedstadt, Germany; 7 KINSYS GmbH, Holtzstr. 2, 76135 Karlsruhe, Germany; 8 LIFE and BRAIN GmbH, Venusberg-Campus 1, Building 76, 53127 Bonn, Germany; 9 Federal Institute for Drugs and Medical Devices (BfArM), Kurt-Georg-Kiesinger-Allee 3, 53175 Bonn, Germany; 10 Department of Nuclear Medicine, School of Medicine, Technical University Munich (TUM), University Hospital Klinikum Rechts der Isar and Central Institute for Translational Cancer Research (Transla TUM, Einsteinstr. 25, 81675 Munich, Germany; 11 Institute for Advanced Study, Department Chemie, Technical University Munich (TUM), Lichtenbergstr. 4, 85747 Garching, Germany; 12 German Center for Cardiovascular Research (DZHK), partner site Heidelberg, 69120 Heidelberg, Germany; 13 Present Address: AskBio GmbH, Am Taubenfeld 21, 69123 Heidelberg, Germany

**Keywords:** Biological techniques, Biotechnology, Cell biology, Neuroscience, Stem cells, Molecular medicine

## Abstract

Human brain cells generated by in vitro cell programming provide exciting prospects for disease modeling, drug discovery and cell therapy. These applications frequently require efficient and clinically compliant tools for genetic modification of the cells. Recombinant adeno-associated viruses (AAVs) fulfill these prerequisites for a number of reasons, including the availability of a myriad of AAV capsid variants with distinct cell type specificity (also called tropism). Here, we harnessed a customizable parallel screening approach to assess a panel of natural or synthetic AAV capsid variants for their efficacy in lineage-related human neural cell types. We identified common lead candidates suited for the transduction of directly converted, early-stage induced neural stem cells (iNSCs), induced pluripotent stem cell (iPSC)-derived later-stage, radial glia-like neural progenitors, as well as differentiated astrocytic and mixed neuroglial cultures. We then selected a subset of these candidates for functional validation in iNSCs and iPSC-derived astrocytes, using shRNA-induced downregulation of the citrate transporter SLC25A1 and overexpression of the transcription factor NGN2 for proofs-of-concept. Our study provides a comparative overview of the susceptibility of different human cell programming-derived brain cell types to AAV transduction and a critical discussion of the assets and limitations of this specific AAV capsid screening approach.

## Introduction

Ever since the groundbreaking introduction of the induced pluripotent stem cell (iPSC) reprogramming technology by Kazutoshi Takahashi and Shinya Yamanaka^1,2^, the field of cell programming has constantly evolved and given rise to a plethora of different techniques, which can now be used to derive, for instance, brain cells for biomedical applications. These techniques comprise strategies using morphogens and/or transcription factors to differentiate iPSCs into the desired cell types^3^, as well as direct conversion approaches enabling transdifferentiation of one somatic cell type into another without a pluripotent intermediate^4^. Next to their use as human cellular platforms for disease modelling^5^, cell programming-derived human cells are promising donor sources for cell replacement strategies^6^. Clinical benefit may also be achieved directly in vivo, for instance, through the conversion of brain-resident glial cells into neurons^7,8^. In that regard, the implementation of efficient and safe-in-human transgene delivery methods represents a crucial prerequisite for further advancing cell programming approaches toward clinical application.

Recombinant adeno-associated viruses (AAVs) are well suited transgene delivery systems for cell programming and engineering, owing to a number of unique assets, including their high versatility^9,10^. The latter is based on the availability of a wealth of natural or synthetic AAV capsids, which, combined with the ease of AAV DNA and protein engineering, offers a great assortment of AAV variants suitable for biomedical applications^11,12^. Notably, swapping the AAV capsid (‘pseudotyping’) can affect both, transduction efficiency and cell type specificity^13,14^, and the outcomes can also differ between species^15^. This provides the option to screen capsid pools in vitro or in vivo, and to identify the best AAV variant for a given species and organ or cell type of interest. For example, in a previous study, human embryonic stem cells (ESCs) and iPSCs, as well as ESC- and iPSC-derived cardiomyocytes were transduced with AAVs of four different serotypes (AAV1, AAV2, AAV6 and AAV9) at three different MOIs (multiplicity of infections). Despite the observation of a significant degree of cell death in undifferentiated cells and much higher absolute transduction efficiencies in differentiated cells, serotypes AAV2 and AAV6 performed best across groups. In a direct comparison of transduction efficiencies of these two AAV variants to lentiviral and adenoviral vectors expressing enhanced green fluorescent protein (EGFP), the adenoviral vectors were superior in undifferentiated cells, whereas ESC- and iPSC-derived cardiomyocytes were best transduced with the tested AAVs^16^. More recently, transduction efficiencies of 11 different AAV capsid variants (AAV1 through AAV9, AAV7m8 and AAV8b) were assessed in human iPSCs, iPSC-derived retinal pigment epithelium and human iPSC-derived as well as rat primary cortical neurons. Measurement of AAV-mediated EGFP expression after transduction at three MOIs and four different time points showed that AAV7m8 performed best across all cell types and MOIs analyzed. Notably, substantial differences in absolute expression levels and time of expression onset were observed between species and cell types^15^.

These and other studies demonstrate that AAV tropism and means for the identification of ideal capsids, as well as transgenes and delivery routes, are pivotal for the success of AAV applications in neural cell types^17^. Curiously, despite significant advances in technologies for directed molecular AAV capsid evolution over the last two decades^11,18^, systematic comparative studies of the increasing numbers of available AAV variants remain scarce. To tackle this shortcoming, some of us have recently established pre-arrayed pan-AAV peptide display libraries in 96-well format and demonstrated the capacity of this platform to rapidly and efficiently screen AAV variants in over 90 transformed or primary cell types of different species^19^. Here, we employed this customizable parallel approach to screen for AAV variants yielding high transduction efficiencies in lineage-related human neural cell types. We specifically included directly converted and iPSC-derived neural stem cells (NSCs) of distinct developmental stages, neurons and/or astrocytes derived thereof, and forward programmed neurons. For most cell types, we could identify AAV capsid variants mediating better transduction efficiency than their respective wild-type parental capsid. Furthermore, we verified that the identified lead candidates were suitable for loss- or gain-of-function studies, such as gene knockdown or transgene overexpression, respectively.

## Results

### Identification of lead AAV capsids for transduction of human neural cell types through parallel screening

**Figure 1:**
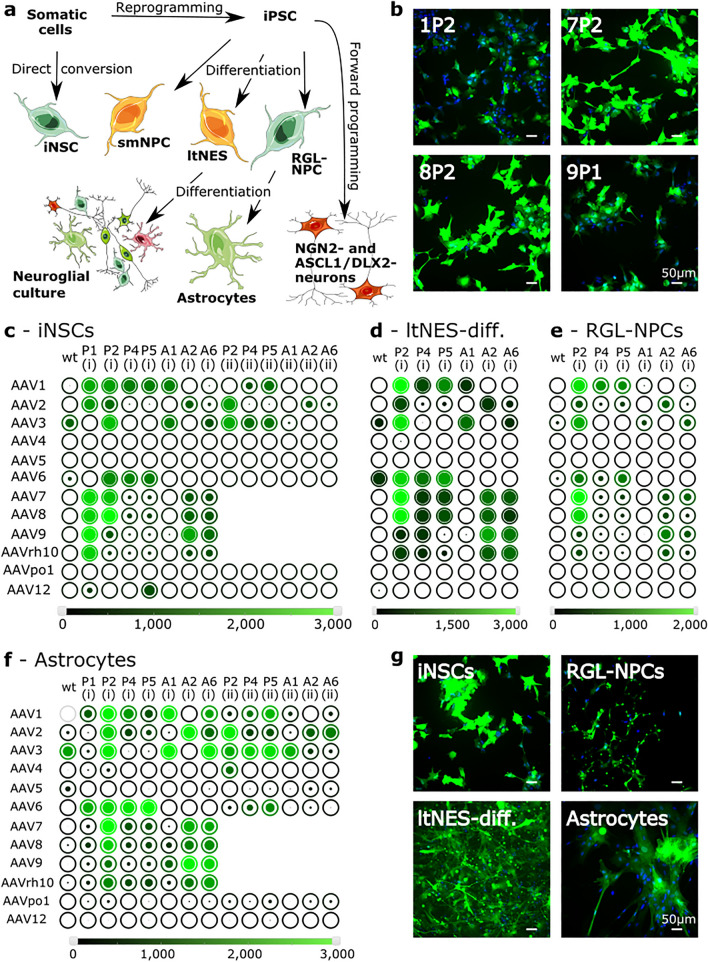
Use of pre-arrayed AAV screening panels to identify lead candidates for efficient transduction of human cell programming-derived neural cell types. (**a**) Schematic representation of the variety of cell programming-derived human cell types tested in this study, as well as their amenability to AAV transduction with the variants used in this screen (green, orange and red colors indicate good, intermediate and poor transducibility, respectively). Components of the figure were adapted from Servier Medical Art (https://smart.servier.com). (**b**) Representative immunofluorescence images of iNSCs after transduction with the AAV variants AAV1P2, 7P2, 8P2 and 9P1. Fluorescence micrographs are depicted at the same gain to visualize differences. (**c**–**f**) Bubble charts representing transduction efficiencies (circle filling; in %) and absolute expression levels (color scalebar; mean gray value of all cells in the analyzed images) of all screened AAV-YFP variants in directly converted iNSCs (**c**), ltNES-derived differentiation cultures (ltNES-diff.) consisting of both, neuronal and glial cells (**d**), iPSC-derived RGL-NPCs (**e**) and RGL-NPC-derived astrocytes (**f**). Each bubble chart is based on the results of a single screening run. wt = wild-type. Inserted peptides (P1, P2, P4, P5, A1, A2, A6) and insertion sites (1, 2) are described in Supplementary Tables S1 and S2, respectively. (**g**) Representative immunofluorescence images of iNSCs, RGL-NPCs, differentiated ltNES cultures comprising neurons as well as astrocytes, and pure iPSC-derived astrocytic cultures after transduction with the lead AAV variant 7P2.

In this study, we harnessed a recently established, customizable parallel screening platform in 96-well format to identify AAV capsid variants that mediate robust transduction efficiency in neural cell types. To this end, we resorted to a subset of our previously reported AAV capsid and peptide collection, which in earlier studies had yielded the most promising results in a diverse collection of transformed and primary cell types from different species^19^. Here, we specifically focused on screening cell programming-derived human neural cell types, which are good proxies for hardly accessible primary human brain cells, and which, thus, are at the center of recent biomedical efforts in the area of disease modelling, drug screening and neuroregeneration. To represent the variety of available cell programming techniques that have been established over the last decades as well as the diversity of human neural cell types and stages, we included the following lineage-related populations in our study (Fig. 1a): (1) Two types of non-polarized, early-stage NSCs derived via either direct cell fate conversion or small molecule-driven differentiation of iPSCs. In the first case, we used induced neural stem cells (iNSCs), which are tripotent *bona fide* NSCs derived from human adult blood cells by direct conversion utilizing overexpression of the two transcription factors SOX2 and cMYC^20^. In the second case, we employed small molecule-derived neural precursor cells (smNPCs), which are self-renewing NSCs generated from iPSCs by using small molecules impacting BMP, TGFβ, GSK3 and SHH signaling^21^. (2) A polarized EGF- and FGF-dependent neural precursor population, which is derived from iPSCs by manual selection and propagation of neural rosettes^22^. Moreover, differentiated neuroglial cultures generated from these so-called long-term neuroepithelial-like stem cells (ltNES) were assessed. (3) Late-stage, radial glia-like neural precursor cells (RGL-NPCs), which are generated from iPSCs via retinoic acid-treated embryoid bodies, and which can be differentiated into almost pure astrocytic cultures due to their strong gliogenic potential^23^. (4) Neuronal cultures derived from iPSCs via transcription factor-mediated forward programming. Specifically, we either overexpressed the neurogenic transcription factor NGN2 alone or a combination of ASCL1 plus DLX2^24^. In addition to these neuroectodermal cell types, we performed AAV screening in iPSC-derived microglia (iPSdMiG), since these mesodermal lineage cells are the immune cells of the central nervous system (CNS), and as such relevant for a number of neurological, neuropsychiatric and neurodegenerative diseases.

All cell types were subjected to AAV screening using a library of AAV capsid variants encoding a yellow fluorescent reporter (YFP) driven by a strong cytomegalovirus (CMV) promoter, and pre-arrayed in a 96-well plate cell culture format. As recently reported by some of us^19^, this technology platform enables the side-by-side comparison of synthetic AAV capsid variants of interest to their respective wild-type AAVs as well as to published AAV benchmarks, using flow cytometry- or microscopy-based measurement of YFP fluorescence as readout for transduction rate (percentage of YFP-positive cells) and expression strength (YFP intensity per cell). Here, we used a particular subset of our reported libraries that comprised 12 AAV wild-types (AAV1 through AAV9, AAVpo1, AAVrh10 and AAV12) as well as an assortment of pan-AAV peptide display variants, i.e, capsids whose tropism has been altered by insertion and display of short, seven or nine amino acids long peptides in an exposed region on the capsid surface (overviews on all introduced modifications and insertion sites are provided in Supplementary Tables 1 and 2, respectively). These selected AAV variants were among the best performers in our recently reported screen on a variety of cell types, including primary murine CNS cells and human astrocytes^19^, and hence deemed suitable and promising for the present work. For screening, cells were plated in 96-well plates and transduced with equal amounts of all AAV capsid variants. Forty-eight hours later, the plates were fixed and subjected to fluorescence measurements using a plate reader and/or microscopy including automated image acquisition and analysis, in order to determine the transduction efficiency and absolute YFP expression of each screened AAV variant. For most cell types employed, single-round screening was performed. However, for iNSCs we exemplarily repeated AAV screening in three additional independent experiments in order to verify the reproducibility of our experimental approach. Both, plate reader analysis of YFP fluorescence and microscopy-based screening readouts of transduction efficiency as well as absolute expression levels demonstrated good reliability (the intraclass correlation for all parameters and readouts was > 0.7), although quantification of imaging data yielded data with higher resolution (Supplementary Fig. S1).

This workflow enabled us to identify several capsid variants that performed well in iNSCs (Fig. 1b,c), ltNES-derived neuroglial differentiation cultures (Fig. 1d), as well as RGL-NPCs (Fig. 1e) and RGL-NPC-derived astrocytes (Fig. 1f). Two genetically distinct smNPC lines and undifferentiated ltNES were also transduced by a subset of AAV variants albeit the YFP expression levels were lower than in the aforementioned cell types. The only cell types that were inefficiently transduced by any of the screened AAV variants were forward programmed NGN2- and ASCL1/DLX2-neurons, as well as iPSdMiG (Supplementary Fig. S2).

Intrigued by this finding, we next set out to investigate whether neurons were transduced within ltNES-derived mixed neuroglial cultures, or whether this cell type might be generally resistant to AAV transduction. To this end, we first stained our screening plate against the pan-neuronal marker TUBB3 48 h after transduction, and were indeed able to identify TUBB3/YFP-double-positive cells (Supplementary Fig. S3). The 96-well screening could not be used to characterize YFP-expressing cells in ltNES-derived mixed neuroglial cultures in greater depth using a more comprehensive marker panel though, because the AAV-YFP screening already occupied three fluorescence channels, which is why only one marker could additionally be stained at a time per screening plate. Therefore, these cells were next seeded in 3.5 cm staining dishes and transduced with a pool of three GFP-encoding AAVs (AAV1P2, 7P2 and 8P2). These AAV variants were identified as lead candidates for the transduction of differentiated ltNES in our previously performed screen. Accordingly, in analogy to the conducted screening, cells were fixed 48 h after transduction and stained for markers of NSCs, glial cells and differentially mature neurons. Quantification of immunofluorescent stainings revealed that AAVs transduced especially NSCs and immature TUBB3-positive neurons in this mixed neuroglial culture, whilst transduction efficiencies decreased in more mature MAP2-positive neurons and astrocytes (Supplementary Fig. S3).

In all robustly transduced cell types (Fig. 1c–f), we found peptide-modified AAV capsid variants that surpassed their respective wild-type capsid in terms of transduction efficiency, including serotypes AAV1, AAV2 or AAV6 that frequently perform well in a wide range of different cell types^19^. More specifically, our best hits comprised serotypes AAV1 and AAV6, whose capsid proteins only vary from each other in a few amino acids^25^, modified with peptides P2, P4 and P5, as well as serotypes AAV7, AAV8, AAV9 and AAVrh10 modified with peptides P2, A2 and A6. Amongst the shared hits, AAV7P2 was the most promising lead candidate as it efficiently transduced iNSCs, ltNES-derived differentiation cultures, RGL-NPCs and RGL-NPC-derived astrocytes (Fig. 1g).

Remarkably, especially the P2 peptide modification greatly increased the transduction efficiency of all screened serotypes except for AAV4, AAV5, AAVpo1 and AAV12. Since the P2 peptide sequence CDC*RGD*CFC includes an RGD motif known to be involved in integrin binding^26–28^, we followed up on this observation using our previously published RNA sequencing data set^20^ to profile expression levels of integrin protein family members in iNSCs, smNPCs and blood cells. As iNSCs were robustly transduced by P1 (*RGD*LGLS)- and P2 (CDC*RGD*CFC)-displaying AAV variants, we further used qPCR to assess the expression levels of *ITGA6*, *ITGAE, ITGB1*, *ITGB5* and *ITGB8*, which were the most strongly expressed integrins in iNSCs (Supplementary Fig. S4). We were specifically interested in comparing expression levels of well-transducible (iNSCs, ltNES-derived differentiation cultures, RGL-NPCs, astrocytes) and difficult-to-transduce (smNPCs, ASCL1/DLX2-neurons, iPSdMiG) cell types. Indeed, we observed a trend toward higher *ITGB8* expression in well-transducible cell types, whereas no evidence for a correlation was detected for the other integrins investigated. To explore the relevance of *ITGB8* for AAV transduction with P1 and P2 peptide variants, we co-treated iNSCs with concentrated AAV7P2- or AAV9P1-YFP and a selective integrin αvβ8 inhibitory ligand. Interestingly, co-incubation of the αvβ8 inhibitor did not affect the fraction of YFP-positive cells for neither AAV variant. However, we noted that higher concentrations of the αvβ8 ligand led to a dose-dependent decrease of the fluorescence intensity in particular in AAV9P1-YFP-transduced iNSCs (Supplementary Fig. S5).

### Validation of the ability of selected AAV capsid variants to modulate gene expression in human neural cells

**Figure 2:**
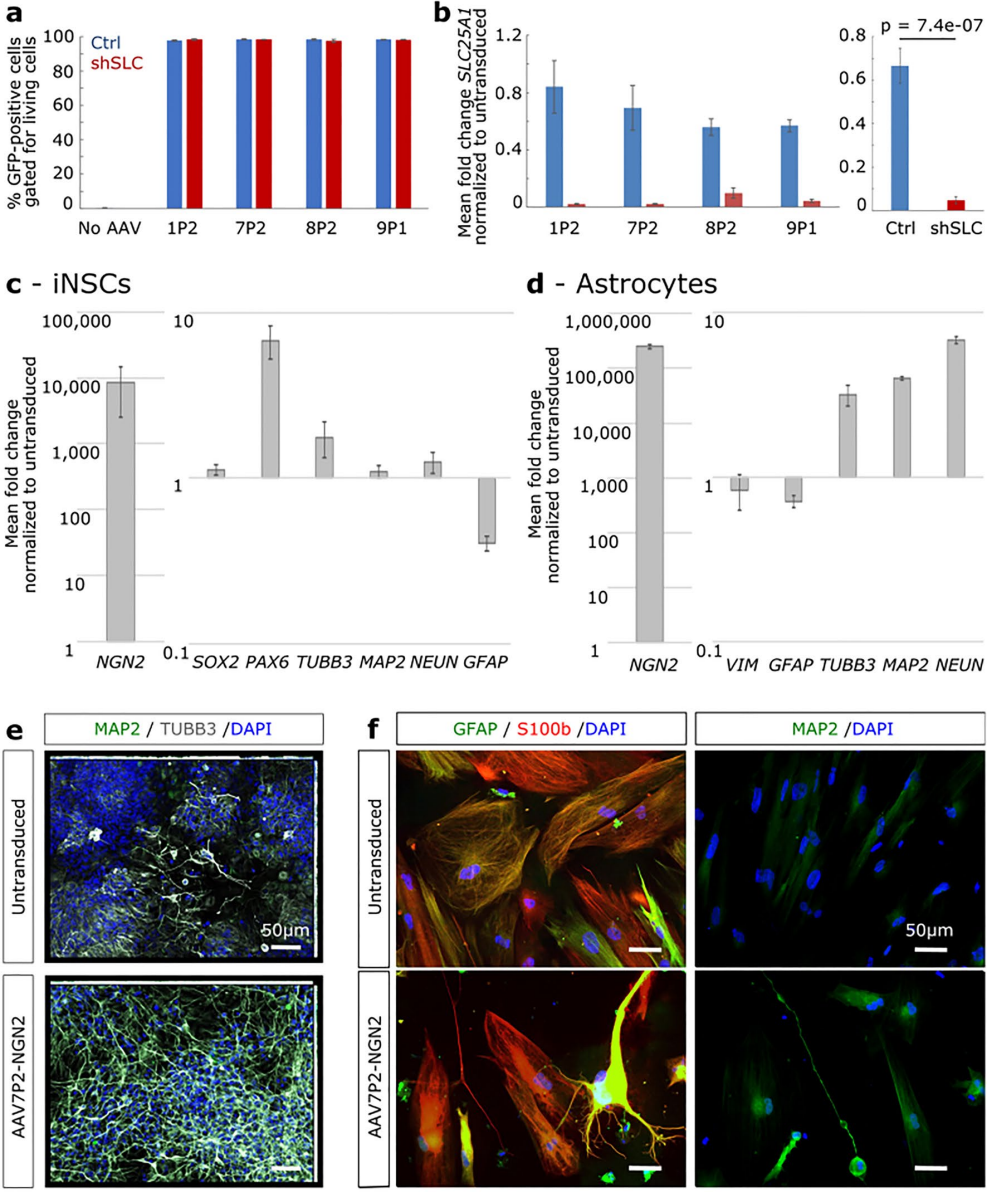
AAVs selected from the screen are capable of achieving shRNA-mediated gene knockdown and transgene overexpression in iNSCs and astrocytes. (**a**) Transduction efficiencies in iNSCs with crude lysates of the lead candidate AAV variants AAV1P2, 7P2, 8P2 and 9P1 expressing GFP along with shRNA-SLC25A1 (shSLC) or a non-silencing control shRNA (Ctrl). Bar graph represents means ± standard error from N = 3 independent experiments per condition. (**b**) Expression of *SLC25A1* as measured by qPCR. Bar graph represents means ± standard error from N = 3 independent experiments per condition. The graph’s left panel depicts the individual data of all AAV variants tested here, whilst the panel on the right depicts the means of all Ctrl and shSLC vectors. Wilcoxon rank sum test was performed on the pooled dataset. (**c**, **d**) qPCR-based expression profiling of neuronal and glial markers after fourteen days of AAV7P2-mediated NGN2 overexpression in spontaneously differentiating iNSCs (**c**) and iPSC-derived astrocyte cultures (**d**). Bar graphs represent means ± standard error of N = 4 from 2 independent experiments. (**e**, **f**) Exemplary images of immunofluorescence stainings against neuronal and/or glial markers in NGN2-transduced and untransduced, differentiating iNSCs (**e**) and iPSC-derived astrocyte cultures (**f**).

We next aimed to verify the potential of selected lead candidates identified in our screen by employing them for loss- or gain-of-function studies. To this end, we first chose the four lead AAV variants AAV1P2, 7P2, 8P2 and 9P1 to explore their efficiency in a knockdown scenario in human iNSCs. As an example, we targeted SLC25A1, which is a mitochondrial carrier exchanging citrate for malatate and as such relevant for the production of acetyl CoA, one of the key players in cell metabolism^29,30^. We employed equal amounts of crude lysates (i.e., non-purified extracts from cells transfected with all plasmids required for AAV production) of these four AAV variants expressing GFP and shRNA targeting SLC25A1 (shSLC) or a non-silencing control (Ctrl). Forty-eight hours after transduction, flow cytometry revealed that all tested variants transduced iNSCs with very high efficiencies ranging from 97.4 to 98.4% (Fig. 2a). Furthermore, in shSLC-transduced cultures, *SLC25A1* expression was nearly abolished on RNA level as compared to controls (Fig. 2b).

In a second example, we used purified and concentrated AAV7P2 vectors to overexpress the neurogenic transcription factor NGN2 in iNSCs and astrocytes. We expected NGN2 overexpression to promote neuronal fate acquisition, since it was shown that overexpression of this transcription factor alone suffices to induce rapid neuronal fate acquisition of iPSCs in vitro^31^ and even converts glia into neurons in vivo^32^. In both cell types investigated, *NGN2* expression levels were highly elevated, i.e., roughly 8500-fold after transduction of iNSCs (Fig. 2c) and around 252,000-fold after transduction of astrocytes (Fig. 2d), as compared to the respective untransduced controls. As expected, AAV7P2-mediated overexpression of NGN2 biased spontaneous differentiation of iNSCs toward a neuronal fate over a period of two weeks. This was evident, for example, from the almost seven-fold elevated expression of *PAX6*, a master regulator of later stage NSC proliferation and neurogenesis, and a known inducer of NGN2^33–40^. Furthermore, expression levels of the neuronal markers *TUBB3*, *MAP2* and *NEUN* were increased, whilst the astrocyte marker *GFAP* was downregulated upon NGN2 overexpression (Fig. 2c). This was further supported by immunofluorescence studies, which showed that NGN2-overexpressing iNSC cultures displayed more prominent expression of the neuronal markers TUBB3 and MAP2 (Fig. 2e). Along the same line, NGN2 overexpression in iPSC-derived astrocytes resulted in a three- to seven-fold increase in the expression levels of the neuronal markers *TUBB3*, *NEUN* and *MAP2* on bulk RNA level (Fig. 2d). This sufficed to initiate cell fate conversion in a fraction of the cultured astrocytes, as single MAP2-positive cells with neuronal morphology were detectable two weeks after AAV7P2 transduction (Fig. 2f).

Collectively, these proof-of-principle experiments demonstrate the power of our parallel screening approach in 96-well format to identify synthetic AAV capsid variants that can be employed to modulate gene expression in different human programmed CNS cell types.

## Discussion

Here, we have capitalized on our recent establishment of a pre-arrayed parallel AAV screening pipeline^19^ and used this to identify AAV capsid variants with high transduction efficiency in diverse human cell programming-derived CNS cell types. Our two major findings are that most of the cell types screened are highly amenable to AAV transduction, and that our parallel screening format can identify lead candidates, which convey better transduction than their parental wild-type serotypes.

Notably though, some cell types, including forward programmed NGN2- as well as ASCL1/DLX2-neurons, were not efficiently transduced by our specific AAV panel. Possible explanations are that these cell types are generally resistant to AAV transduction or require a higher AAV dose than what is provided by the non-concentrated crude lysates in our pre-arrayed screening plates. In this context, it is interesting to note that, although purified cultures of forward programmed neurons were not efficiently transduced by our AAV screening panel, immunofluorescence analysis of AAV-transduced ltNES-derived neuroglial cultures revealed that especially NSCs and less mature human neurons are, in principle, amenable to AAV transduction. Our results are overall in line with previous data showing that in both, iPSC-derived as well as primary human cell cultures, AAV9P1-CMV-EYFP (a peptide display variant of AAV9 that was also included in the present panel) yields lower transduction rates in neurons as compared to astrocytes^14^. Moreover, it has been reported that transduction efficiencies of 11 different AAV variants are much lower for human iPSC-derived than rat primary cortical cultures^15^, which also supports the notion that human neurons are difficult to transduce with AAVs. Interestingly, in this published screen, AAV-mediated EGFP expression from an eCMV and chicken beta-actin (CBA) promoter-driven construct in human iPSC-derived neurons was first detected at 96 h after transduction with the highest MOI tested (10^6^ viral genomes (vg)/cell)^15^. Thus, the poor neuronal transduction rates observed in our forward programmed neuron screens could be due to low virus concentrations in the crude lysates, a too short follow-up (48 h post-transduction) in this screen and/or a too advanced neuronal maturation state acquired by forward programmed neurons at the time of AAV transduction. These findings are concordant with others, altogether indicating that transduction patterns in mature neurons are species-specific and could, at least in vivo, potentially even depend on the age of an organism at the time of AAV treatment^17^.

In addition to these limitations, it is key to realize that the pivotal benefits of our screening platform, i.e., its speed and ease, come at the prize of a limited capsid and promoter diversity in the original panel. Unlike a conventional directed evolution approach that typically starts with a complex library of millions of distinct AAV variants, which is then iteratively screened on target cells over multiple rounds of infection and rescue, our current approach is based on a much smaller library of pre-selected capsid variants encoding an expression cassette composed of the CMV promoter driving the fluorescent reporter EYFP, thus enabling a rapid read-out after a single screening round. Of all available options, we decided to use the CMV promoter as it is known for its rather ubiquitous, strong and rapid expression, which benefitted our experimental screening setup in which we monitored fluorophore expression 48 h after transduction of various different cell types. It is well possible that the cell lines that came back negative in our current screens might show EYFP expression with other promoters or AAV capsid variants lacking in our current panel. For instance, use of Synapsin promoter-driven constructs in an AAV capsid library could improve the screening in more mature forward programmed neuronal cultures. As for iNSCs, our follow-up experiments show that constructs driven by an EF1α promoter can also be functionally transduced by AAVs. Nevertheless, systematic screening approaches in lineage-related cell types, such as the one employed in this study, could be valuable as a basis to select the most promising AAV variants for subsequent studies aiming at further enhancing AAV performance in a model-orientated manner. For example, directed evolution was previously used to generate an AAV2/AAV5 chimera with an A581T point mutation, which is highly specific to and infectious in an organotypic human airway model^41^. Similar studies have already been conducted in vitro for human pluripotent stem cells^42^ and rat primary as well as human ESC-derived neural precursor cells^43^, and even in vivo targeting different mouse brain cell types^44^.

Considering the inherent trade-off of the pros and cons of this particular parallel AAV screening strategy, it is noteworthy that the ability to compare a custom-built selection of AAV variants across various cell types could also be valuable for identifying, for instance, AAV cell entry mechanisms that might be common for several capsid variants in lineage-related cell types. In this regard, recurrent patterns in capsid modifications yielding increased transduction efficiencies could be particularly relevant. The peptides we here identified to yield good transduction efficiencies in a number of cell types can be roughly grouped into two classes: While peptides P1 and P2 carry an RGD (*RGD*LGLS and CDC*RGD*CFC, respectively) motif, peptides P4, A2 and A6 are commonly characterized by a NXXR (*NDVR*SAN, *NYSR*GVD and *NEAR*VRE, respectively) motif. Some of us and numerous other groups have previously isolated such NXXR-containing peptides in different AAV backbones in divergent cell types ex vivo and in vivo^19,45–50^. This suggests that rather than interacting with an external cellular receptor that would have to be shared by all these distinct target cells, these peptides improve intracellular steps in AAV transduction that are receptor-independent. This hypothesis is supported by data from Naumer et al*.*^45^ who showed that in the AAV2 background, the P4 peptide primarily acts intracellularly downstream of AAV entry. RGD motifs, on the other hand, are thought to govern AAV cell attachment and entry via binding to various integrins on the cell surface. For this reason, we have focused on the RGD-containing P1 and P2 variants in our study. Preliminary data obtained in the current work could indeed suggest that integrin binding might represent a promising target for future studies aiming at optimized transduction. In line with this idea, a recent paper described the superiority of RGD motif-containing AAVs for the transduction of myocytes in mice and non-human primates^51^, concurrent with data reported before by some of us for the RGD-displaying AAV9P1 variant in the musculature of mice^52^. However, the relation between integrin (subtype) expression and AAV biology is likely to be very complex^53,54^. For instance, while our experiments revealed a prominent decrease of fluorescent intensity when iNSCs transduced with AAV9P1-YFP were treated with higher concentrations of a selective integrin αvβ8 ligand, the overall fraction of transduced cells appeared unchanged. These findings call for further mechanistic studies into the connection between cell type-specific AAV variant tropism and integrin expression patterns.

It has to be further acknowledged that, while increasing the number of AAV variants that can be assessed in parallel, our current experimental readout limits the mechanistic insight that can be obtained, as it resorts to the quantification of reporter transgene expression as a measure of functional transduction efficiency. In future studies following up on pre-selected AAV lead candidates, it might be interesting to relate functional transduction efficiency to molecular transduction rates, which could, for example, be determined by recovering genome copies post-transduction with concentrated AAVs. Such an approach might reveal interesting additional information on the biology of distinct AAV variants, as it would further allow to compare potential differences in intracellular processing and signal amplification across cell types and AAV variants.

Lastly, we demonstrate that top hits identified by parallel AAV screening not only excel at the transduction of a reporter gene, but can also be harnessed to functionally modulate gene expression in neural cells. For example, we identified and verified the AAV peptide display variants AAV1P2, 7P2, 8P2 and 9P1 as lead candidates for the transduction of human iNSCs. These results are in line with one of our previous studies where 30 AAV variants — which are also included in the current screening panels — were used for the transduction of HNSC.100 cells kept as proliferating NSCs or differentiated astrocyte-enriched cultures, and which identified AAV9P1 as the variant yielding the highest transduction efficiencies^14^.

Importantly though, one remaining key question is whether and to what extent screening results obtained in simplified 2D in vitro systems can predict in vivo functionality and thus clinical translatability. Here, it is worth noting that the wild-type form of AAV9 — which was the first AAV serotype described to be capable of bypassing the blood–brain-barrier^17 ^— or a capsid-modified variant thereof (i.e., AAV9P1) have already been used in vitro in more complex, iPSC-derived 3D organoid models^14,55^, as well as in vivo to treat mice suffering from either ischemic brain injury^56^ or Huntington’s disease^57^ by mediating astrocyte-to-neuron cell fate conversion. On top of this, two studies recently published by some of us illustrate that the screening approach employed here is, in principle, applicable in vivo when combined with molecular barcoding and next-generation sequencing^52,58^. For instance, injecting 177 barcoded AAV-YFP variants (representing a library that is significantly overlapping with the one used in this study) into the lateral ventricle of adult mice enabled the identification of two lead candidates, namely AAV variants 9A2 and 1P5, capable of efficiently transducing NSCs in the ventral subventricular zone^58^.

It is interesting to note though, that AAV9A2 and 1P5, which some of us identified as lead candidates in this recent study in endogenous mouse NSCs^58^, were outperformed by other capsid variants in human iNSCs and iPSC-derived RGL-NPCs in the present work. This notion again stresses the cell type- and species-specificity of AAV transduction and the ensuing need to always evaluate the performance of available AAV variants within the proper context, for instance by means of parallel capsid screening as exemplified here. Along this line, it will eventually be interesting to explore how our AAV screening results in human cells translate to an in vivo scenario. Since it was recently shown that capsid-promoter interactions can significantly impact AAV tropism in vivo^59^, it can be foreseen that the customizable combination of capsid-modified AAV lead candidates with selective promoters and/or miRNAs may help to increase transduction efficiency and cell type-specificity that can be achieved by AAV variants in such a context. This might also facilitate targeting one out of multiple cell types present in more complex CNS models such as cerebral organoids or even in native brain tissue.

## Methods

### Cell culture

All human cell programming-derived neural cell types were generated, cultured and/or expanded according to previously described methods: iNSCs^20^, smNPCs^21^, ltNES^22^, RGL-NPCs^23^, as well as forward programmed NGN2- and ASCL1/DLX2-neurons^24^. IPSdMiG were generated via a proprietary protocol from LIFE and BRAIN GmbH (patent application number EP20162230). Details are found in the Supplementary Methods.

### AAV production and transduction

AAV vectors were produced in HEK293T cells using a standard triple-transfection protocol and an AAV helper plasmid encoding AAV *rep* and *cap* genes, an AAV vector plasmid carrying the transgene as well as an adenoviral helper plasmid^60^. Wild-type *cap* genes were modified through insertion of DNA oligonucleotides encoding 7mer or 9mer peptides whose sequences and properties were reported^19^ and are provided in Supplementary Table S1. For some of the employed AAV serotypes, we have included two different versions with two distinct neighboring peptide insertion sites based on primary sequence alignment and 3D structure predictions, as reported in detail in Börner et al.^19^. In the past, we had observed different phenotypes of these permutations in various other target cells, although without a clear pattern that would consistently identify one of the two sites as superior. For that reason, we have also tested both permutations per capsid in the current study to ensure that we would not miss a promising candidate.

For parallel AAV capsid screening in 96-well plate format, crude cell lysates from triple-transfected 6-well plates containing non-purified AAV particles were used. Therefore, 10 µl AAV were added per well with a multichannel pipette directly into the medium (200 µl) of the pre-plated cells (1:20 dilution). For the characterization of ltNES-derived neuroglial differentiation cultures and AAV-mediated gene knockdown, cells were cultured in 3.5 cm dishes and transduced with crude lysates at a 1:20 dilution.

For purification by iodixanol gradient density centrifugation, cells were collected by scraping 72 h after transfection, followed by centrifugation at 500× *g* for 15 min at room temperature. The cell pellet was resuspended in 20 ml Benzonase buffer and lysed by five freeze–thaw cycles in liquid nitrogen and a 37 °C water bath. Cell debris was sonicated for 1 min and subjected to two centrifugations steps, each at 4000× *g* for 15 min at 4 °C. The virus-containing lysate was purified via iodixanol density gradient ultracentrifugation (290,000× *g*, 120 min, 4 °C) and collection of the 40% phase. This AAV-containing fraction was rebuffered in 1xDPBS using amicon spin columns (Merck, Darmstadt, Germany) and AAVs were stored in aliquots at − 80 °C.

Viral genome titers were determined by qPCR as described in Senís et al*.*^10^ using primers and probes for CMV, or by utilizing the AAV quantification kit from Norgen Biotek Corporation (Ontario, Canada). For integrin αvβ8 inhibition and NGN2 overexpression, concentrated AAVs were used at a dose of 2 × 10^6^ vg/cell and 6 × 10^4^ vg/cell, respectively. The production and characterization of the selective integrin αvβ8 ligand [*c*(GLRGDLp(NMe)KAc], which was dissolved in DMSO and co-applied to iNSCs along with concentrated AAV7P2-YFP or AAV9P1-YFP for 48 h, is described in detail elsewhere^61,62^. Notably, this ligand shows low nanomolar binding affinity to αvβ8 (8.0 nM), in contrast to αvβ6 (99 nM), αvβ3 (343 nM), αvβ5, α5β1 and αIIbβ3 (all > 1,000 nM)^61,62^.

### Cloning procedures

To generate an AAV vector expressing an shRNA against SLC25A1, two complementary oligonucleotides were used, each comprising the sense (5′-GCCATCCGCTTCTTCGTCATGA-3′) and antisense strand (5′-TCATGACGAAGAAGCGGATGGC-3′), as well as the central loop sequence TCAAGAG. The 5’ ends were flanked by the sequence CACC on the forward strand and by AAAA on the reverse strand. Following annealing, these flanking sequences produced overhangs that were used for direct ligation using a Golden Gate protocol into the BbsI-digested AAV vector plasmid TRISPR^63^ (details of this plasmid will be reported elsewhere).

To clone the pAAV-EF1α-NGN2-3xFLAG expression vector, plasmids pAAV-EF1α-NGN2-RFP-NLS (details of this plasmid will be reported elsewhere) and NR6AI-C-term-3xFLAG (kindly provided by Nils Braun) were used as templates. The NGN2 and 3xFLAG sequences in these plasmids were PCR-amplified with appropriate overhangs for cloning using primers 5′-TGTCGTGAGGAATTTCGACGGATCCGCCACCATGTTCG-3′ plus 5′-CTTTATAATCTTTATCATCATCATCTTTATAATCGATACAATCCCTGGCTATGG-3′ (for NGN2) or 5′-CCATAGCCAGGGATTGTATCGATTATAAAGATGATGATGATAAAGATTATAAAG-3′ plus 5′-TTCAACATGCCTCCGCCCTTATCATTTATCATCATCATCTTTATAATC-3′ (for 3xFLAG). Gibson assembly was performed using the 2× Gibson assembly master mix (New England Biolabs, Frankfurt am Main, Germany). Thermo Fisher Scientific’s (Waltham, USA) plasmid DNA Miniprep and Maxiprep kits were used for DNA isolation.

### Immunocytochemistry

Forty-eight hours after transduction with the AAV panels, cells were washed once with 1×DPBS before fixation with 4% PFA for 10 min at room temperature. Afterwards, cells were washed once with 1×DPBS before incubation with 2 µg/ml DAPI for 5 min at room temperature. DAPI was replaced by 200 µl 1×DPBS per well before YFP fluorescence was measured using an Infinite M Plex plate reader (Tecan Group, Männedorf, Switzerland; at 500 nm excitation and 540 nm emission) and/or immunofluorescence image acquisition and analysis (see below). For all other immunofluorescent stainings, blocking with 0.5% Triton-X and 10% FBS was performed after fixation, followed by overnight incubation with primary antibody solution at 4 °C (1:1,000 rabbit GFAP from Merck Millipore, Burlington, USA, Cat. AB5804; 1:100 rabbit MAP2 from Abgent, San Diego, USA, Cat. AP2018E; 1:200 rabbit Nestin (NES) from Bio-techne, Minneapolis, USA, Cat. NB300-265; 1:1,000 mouse S100b from Sigma-Aldrich, St. Louis, USA, Cat. S2535; 1:100 mouse SOX2 from R&D systems, Minneapolis, USA Cat. MAB2018; 1:400 chicken TUBB3 from Merck Millipore, Cat. AB9354; 1:1,000 mouse TUBB3 from Eurogentec, Lüttich, Belgium, Cat. MMS-435P; 1:1,000 rabbit TUBB3 from biolegend, San Diego, USA, Cat. 802001; in 0.3% Triton-X and 5% FBS). The next day, three washing steps with 0.3% Triton-X were performed before secondary antibody solution was incubated for 2 h at room temperature (1:500 donkey-anti-rabbit AF-647, Cat. A31573; 1:500 goat-anti-chicken AF-647, Cat. A21449; 1:1,000 goat-anti-mouse AF-555, Cat. A21424; 1:500 goat-anti-mouse AF-647, Cat. A21236; 1:1,000 goat-anti-rabbit AF-488, Cat. A11008; 1:1,000 goat-anti-rabbit AF-555, Cat. A21429; 1:500 goat-anti-rabbit AF-647, Cat. A21244; in 0.3% Triton-X and 5% FBS; all Thermo Fisher Scientific). After three additional washing steps, nuclei were counter-stained with DAPI. Wells were sealed with Mowiol plus DABCO (Carl Roth, Karlsruhe, Germany).

### Image acquisition and analysis

Live-cell phase contrast and fluorescence images were acquired with a PAULA cell imager (Leica Microsystems, Wetzlar, Germany). Automated image acquisition of 96-well plates was performed on an epifluorescence Scan^R screening microscope (Olympus Biosystems, Münster, Germany) equipped with the Scan^R acquisition software (version 2.4.0.13 for scan^R 2.4; © 2004–2013 Olympus Soft Imaging Solutions GmbH) or an IN Cell analyzer 2200 (GE Healthcare Bio-Sciences Corp, Piscataway, USA) with the respective IN Cell analyzer 2200 software (version 6.2-15347; 64-bit; December 12, 2016). 10× and 20× objectives were used with appropriate excitation and emission filters to measure 9 and 16 positions per well, respectively. For AAV screening, Scan^R images were subsequently processed with a previously established pipeline for quantitative image analysis^64^. This yielded the mean gray value of all cells in the analyzed images, total cell counts, and percentages of AAV-positive cells (determined via fluorescent reporter). Non-quantitative image processing of automatically acquired IN Cell images for qualitative display was performed with defined CellProfiler (version 2.2.0^65^, © Broad Institute, Cambridge, USA; https://www.cellprofiler.org) pipelines running the ‘ApplyThreshold’ (threshold strategy: manual) and ‘RescaleIntensity’ (rescaling strategy: divide each image by the same value) modules. Alternatively, images were manually captured with an AxioImager plus Apotome (Carl Zeiss, Oberkochen, Germany) and the AxioVision software (version 4.8.2.0; © 2006–2010 Carl Zeiss Microimaging GmbH). Quantification of manually acquired images was performed using a semi-automated, three-step pipeline specifically developed in the context of this project (deposited on github: https://github.com/simon-ziegler/cell-segmentation). (1) Cell segmentation**:** First, single cells and cell clusters were segmented using the DAPI channel. The segmentation was primary based on adaptive thresholding and Gaussian filtering. Objects were distinguished between invalid objects, cells and cell-clusters depending on size and shape. Cell clusters were broken up into cells by creating distance maps and their local maxima, which were used as the starting point of a watershed algorithm. (2) Background separation: Second, after an initial step of intensity normalization based on the maximum intensity, foreground and background were separated in all other (marker) channels. This could either be done by a thresholding based on intensity quantiles or by fitting Gaussian curves to the intensity distribution. Alternative to two Gaussians (foreground and background), we also found good results when using three Gaussians to distinguish between background, low- and high-expressing cells for some markers stained. Based on the locations of the Gaussians the separation threshold(s) were defined. (3) Classification: Third, the cells identified in step 1 were automatically denotated as marker-positive or -negative by comparison of their mean intensity in the marker channels with a manually set threshold or by probability assignment, if using the Gaussian-fitting approach from step 2. Further options applied to optimize the categorization process comprised measures of texture homogeneity, entropy or energy of objects. As a final step, automatic object categorization could be manually revised, if necessary. After quantification, images were post-processed with ImageJ (version 1.52a; Java 1.8.0_301; 64-bit; © Wayne Rasband, National Institutes of Health, USA; http://imagej.nih.gov/ij) for display. Image processing included adjustments of brightness and contrast, and was equally applied across single channel pictures.

### Flow cytometry

In order to quantify the percentage of successfully transduced cells and their fluorescence intensity via flow cytometry, iNSCs were detached and singularized by Accutase (Thermo Fisher Scientific) treatment. GFP and YFP fluorescence was measured in FL1 on an BD Accuri ™ C6 Plus flow cytometer (BD Biosciences, San Jose, USA). Events were gated for living single cells and data analyzed with the associated BD Accuri ™ C6 Plus analysis software (version 1.0.23.1; build 20151211.23.1; © 2011 Accuri ® Cytometers, Inc.).

### qPCR

RNA extraction and cDNA synthesis were performed with Qiagen’s (Hilden, Germany) RNeasy and Quanta biosciences’ (Beverly, USA) qScript kit, respectively, according to the manufacturers’ instructions. qPCR was performed on an Eppendorf (Hamburg, Germany) iCycler in 96-well format. Per reaction, 300 ng cDNA were diluted in a total volume of 19 µl qPCR master mix (ddH_2_O with 200 µM of each dATP, dGTP, dCTP and dTTP, 10 nM fluorescin calibration dye, 0.75× SyBr Green, 4% DMSO, 1× PCR Rxn buffer, 3 mM MgCl_2_, and 0.6 U Taq polymerase (Thermo Fisher Scientific)) and supplemented with 1 µl primer mix consisting of 5 µM forward and reverse primers (all IDT, Coralville, USA). Primer sequences were as follows: *18 s* F: 5’-TTCCTTGGACCGGCGCAAG-3’, R: 5’-GCCGCATCGCCGGTCGG-3’; *ITGA6* F: 5’-AAAGCCTGCTCAATCCCTGA-3’, R: 5’-GTAAGTCAGCCACGCCAAAA-3’; *ITGAE* F: 5’-GGTTCTTTCTCCTTGGCACG-3’, R: 5’-CATCTGCCCCAAAACCTTCC-3’; *ITGB1* F: 5’-AGCTTTAAAACCTGTGTGCCAT-3’, R: 5’-CAAAAGGTCCCCATTCAGCAA-3’; *ITGB5* F: 5’-GGCTTGATCACAGCTCCCTA-3’, R: 5’-GGACAACAAGCTGGATGTGG-3’; *ITGB8* F: 5’-AAGAGCAGTCACCTACCGAC-3’, R: 5’-CGTTCGAGTGTACAACCAGC-3’; *GFAP* F: 5’-ATCGAGAAGGTTCGCTTCCT-3’, R: 5’-CAGCCTCAGGTTGGTTTCAT-3’; *MAP2* F: 5’-AAAGAAAAGGCCCAAGCTAAA-3’, R: 5’-GCTTCCAGACGAGGAGACA-3’; *NEUN* F: 5’-GGATGGATTTTATGGTGCTGA-3’, R: 5’-ACATGGTTCCAATGCTGTAGG-3’; *NGN2* F: 5’-CTGGGTCTGGTACACGATTG-3’, R: 5’-GGTTGTTGGCCTTCAGTCTA-3’; *PAX6* F: 5’-AATAACCTGCCTATGCAACCC-3’, R: 5’-AACTTGAACTGGAACTGACACAC-3’; *SLC25A1* F: 5’-CCCCATGGAGACCATCAA-3’, R: 5’-CCTGGTACGTCCCCTTCAG-3’; *SOX2* F: 5’-GTATCAGGAGTTGTCAAGGCAGAG-3’, R: 5’-TCCTAGTCTTAAAGAGGCAGCAAAC-3’; *TUBB3* F: 5’-CCATCTTGCTGCCGACAC-3’, R: 5’-CAATAAGACAGAGACAGGAG-3’; *VIM* F: 5’-TCTCTGAGGCTGCCAACCG-3’, R: 5’-CGAAGGTGACGAGCCATTTCC-3’. qPCR reactions were run in duplicates or triplicates and cycling conditions were as follows: 3 min at 95 °C; 40 cycles of 15 s at 95 °C, 20 s at 60 °C and 30 s at 72 °C; 1 min at 95 °C and 15 s at 55 °C, followed by a temperature gradient to 95 °C over the course of 20 min and finally 15 s at 95 °C, before cooling down to 4 °C. Ct values of the genes of interest were normalized to the house-keeping gene *18 s* and transformed to mean fold changes using the equation 2^−∆∆Ct^. qPCR products were size-validated by gel electrophoresis.

### Statistical analysis

All statistical analyses were performed with R (version 3.5.1; © The R foundation for statistical computing, 2018; https://www.R-project.org). For assessing method reliability, intraclass correlation coefficients were determined in two-way random effects models based on single units. To assess method concordance, a line of identity plot was generated and a concordance correlation coefficient calculated. In all other instances, Kolmogorov–Smirnov’s and Shapiro Wilk’s test for normal distribution, as well as Levene’s test and/or F-test for homogeneity of variance were first performed in order to determine whether variables met the assumptions of linear models. Since data were not normally distributed, two-sided Wilcoxon rank sum tests were calculated to compare two groups. Correlation was assessed using Kendall’s τ. The significance level was set to *p* < 0.05. Multiple testing on the same set of data was not performed.

### Statement of ethical approvals

The collection of human somatic material (i.e., blood or fibroblasts) from healthy donors for iPSC reprogramming and iNSC conversion was in accordance with German law and approved by the ethics committee of the University of Bonn Medical Center (approval number: 275/08). All subjects gave written informed consent.

## Supplementary Information

Supplementary Information.

## Data Availability

The AAV screening data will be deposited in the freely accessible online database SPIRIT (https://spirit.cladiac.com/aav.html) upon publication.
