# Supplementary material for: Identification of adeno-associated virus variants for gene transfer into human neural cell types by parallel capsid screening
Source: Sci Rep. 2022 May 19;12:8356. doi: 10.1038/s41598-022-12404-0 (PMC9120183; doi:10.1038/s41598-022-12404-0)
Supplement: Supplementary file 1 — Supplementary Information. [file 41598_2022_12404_MOESM1_ESM.pdf]

Identification of Adeno-associated virus variants for gene transfer into human neural cell types by parallel capsid screening

Lea Jessica Flitsch, Kathleen Börner, Christian Stüllein, Simon Ziegler, Vera Sonntag-Buck, Ellen Wiedtke, Vesselina Semkova, Si Wah Christina Au Yeung, Julia Schlee, Mohamad Hajo, Mona Mathews-Ajendra, Beatrice Stefanie Ludwig, Susanne Kossatz, Horst Kessler, Dirk Grimm and Oliver Brüstle

Supplementary information

Supplementary Methods

Cell culture

Neural stem cells

Four different populations of human NSCs were included in this study, representing different developmental stages. Directly converted, blood-derived early stage iNSCs[20] were cultured on Matrigel-coated dishes (Corning, Corning, USA, 1:60) in 2x N2B27 (1:1 Advanced DMEM/F12 : Neurobasal (both Thermo Fisher Scientific, Waltham, USA) with 1x N2 supplement (GE Healthcare, Chicago, USA), 1x B27 supplement without vitamin A, 1 mM L-glutamine and 0.0025% BSA (all Thermo Fisher Scientific)) supplemented with 3 µM CHIR99021, 0.5 µM purmorphamine (both Miltenyi, Bergisch Gladbach, Germany), 0.5 µM A83-01 (Tocris, Bristol, UK), 10 ng/ml human LIF (Novoprotein, Fremont, USA) and 64 µg/ml LAAP (Sigma-Aldrich, St. Louis, USA). Early stage iPSC-derived smNPCs[21] were grown on Geltrex-coated dishes (Thermo Fisher Scientific, 1:50) in 1x N2B27 (1:1 DMEM/F12 (Thermo Fisher Scientific) : Neurobasal with 0.5x N2 supplement, 0.5x B27 supplement without vitamin A, 1 mM L-glutamine and 1x Pen/Strep (Thermo Fisher Scientific)) supplemented with 3 µM CHIR99021, 0.5 µM purmorphamine and 64 µg/ml LAAP. For virus screening, $1x10^4$ cells were seeded in 200 µl of their respective medium per well of a 96-well ibidi imaging plate (Gräfelfing, Germany). For AAV and integrin $\alpha v\beta 8$ ligand co-treatment, $2x10^5$ iNSCs were seeded in 500 µl iNSC medium per well of a 24-well plate. For assessment of proliferative and differentiated iNSCs after AAV-mediated shRNA-SLC25A1 and NGN2 overexpression, cells were seeded at a density of $5x10^4$ cells/cm^2 and $9x10^4$ cells/cm^2, respectively. For gain-of-function experiments with iNSCs, spontaneous differentiation was induced by switching culture medium to NGMC at the day of AAV transduction (1:1 DMEM/F12 : Neurobasal with 0.5x N2 supplement, 0.5x B27 supplement without vitamin A, 800 µg/ml D(+)glucose (Carl Roth, Karlsruhe, Germany), 0.5 mM L-glutamine and 1x Pen/Strep, supplemented with 10 ng/ml BDNF, 10 ng/ml GDNF (both Cell Guidance Systems, Cambridge, UK), 64 µg/ml LAAP and 0.5 mM dibutyryl-cAMP (Sigma-Aldrich)).

Polarized ltNES[22] were cultured in N2 medium (DMEM/F12 with 20 nM progesterone, 100 µM putrescin, 30 nM sodium selenite, 100 µg/ml apo-transferrin (all Sigma-Aldrich), 25 µg/ml insulin (Thermo Fisher Scientific) and 1.6 mg/ml D(+)-glucose) supplemented with 0.05x B27 supplement (Thermo Fisher Scientific), 10 ng/ml EGF and 10 ng/ml bFGF (both R&D systems, Minneapolis, USA) on poly-L-ornithine-laminin (Sigma-Aldrich)-coated dishes. $1.3x10^5$ ltNES were seeded in 200 µl medium per well in a 96-well plate for AAV screening.

Later stage iPSC-derived gliogenic RGL-NPCs[23] were cultured on Geltrex-coated dishes (1:50) in N2 medium (DMEM/F12 with 1x N2 supplement, 1.6 mg/ml D(+)-glucose, 25 µg/ml insulin and 1x Pen/Strep) supplemented with 20 ng/ml EGF and 20 ng/ml bFGF. For virus screening, $0.7x10^4$ RGL-NPCs were seeded in 200 µl medium per well in a 96-well plate.

Neurons

Three different types of iPSC-derived neuronal cultures were subjected to virus screening. LtNES were differentiated into neuroglial cultures by withdrawing the growth factors EGF and bFGF. Cells were differentiated in 1:1 DMEM/F12 : Neurobasal with 0.5x N2 supplement, 0.5x B27 supplement, 0.9 mg/ml D(+)-glucose, 10 µg/ml insulin and 1x Anti-Anti (Thermo Fisher Scientific), and cryo-preserved on day 14 of differentiation. For experiments, pre-differentiated cells were thawed in ltNES differentiation medium supplemented with 10 ng/ml BDNF and 10 ng/ml GDNF, seeded at a density of $2.5x10^4$ cells per well for virus screening and $5.5x10^4$ cells/cm^2 for cell type characterization, and further matured up until day 27 of differentiation. Alternatively, iPSCs were differentiated into neurons via overexpression of the transcription factor(s) NGN2 or ASCL1 plus DLX2 according to a previously published protocol.[24] On day 8 of forward programming, cells were replated and seeded at a density of $4.5x10^4$ cells per well. One day after seeding, transduction of the AAV screening panel was performed. For all neuronal cultures, AAV panel transduction was performed in 200 µl medium per well in a 96-well plate.

RGL-NPCs and astrocytes

In order to derive highly purified astrocyte cultures, RGL-NPCs were seeded at a density of $9x10^4$ cells/cm^2 on Matrigel-coated dishes (1:30). Differentiation was initiated by switching to N2 medium supplemented with 100 µg/ml apo-transferrin, 10 ng/ml human LIF and 10 ng/ml BMP-4 (Thermo Fisher Scientific). For virus screening, cells were replated on day 28 of astrocyte differentiation, by seeding $0.7x10^4$ astrocytes per well. Virus screening was performed on days 29-31 of differentiation in 200 µl medium. For functional experiments, astrocytes were seeded at a density of $9x10^4$ cells/cm^2 on day 14 of astrocyte differentiation and AAVs were transduced one week later. Upon AAV transduction, medium was changed to N2 medium (1x DMEM/F12 with 1x N2 supplement, 1.6 mg/ml D(+)glucose, 50 µg/ml insulin, 100 µg/ml apo-transferrin and 1x Pen/Strep) supplemented with 1x B27 supplement without vitamin A, 10 ng/ml BDNF and 10 µM Rock-inhibitor Y-27632 (Cell Guidance Systems), in order to support the survival of astrocytes as well as eventually emerging neurons.

iPSC-derived microglia

To generate human iPSdMiG, iPSCs were cultured on Geltrex-coated 6-well plates (180 µg/ml) in iPS-brew (Stemcell Technologies, Vancouver, Canada) medium and passaged using 0.5 mM EDTA (Sigma-Aldrich). To induce differentiation, embryoid bodies were generated by detaching intact iPSC colonies using 1 mg/ml collagenase (Thermo Fisher Scientific) dissolved in DMEM/F12. Detached colonies were cultured in suspension for four days before being seeded on poly-L-ornithine- plus fibronectin (Sigma-Aldrich)-coated culture plates. The differentiation of iPSdMiG was carried out according to a proprietary protocol of the LIFE & BRAIN GmbH (patent application number EP20162230). In short, neuroepithelial and hemogenic endothelial precursors were differentiated into neural and immature microglial cells within the same culture paradigm. After approximately six weeks of differentiation, mature microglia were released into the supernatant of the multilineage differentiation culture, from which they could be repeatedly harvested and plated in poly-L-lysine (Sigma-Aldrich)-coated cell culture plates in order to obtain homogeneous, adherent cultures. For AAV screening, $2.3x10^3$ harvested iPSdMiG were seeded per well in 200 µl of their respective medium.

Supplementary Tables

Supplementary Table S1: Overview of peptide modifications inserted into the capsids of diverse AAV serotypes. Table adapted from Boerner *et al.*, 2020.[19]

Peptide	Source Reference	DNA sequence [a]	Protein sequence
P1	66	CGC GGC GAT CTG GGC CTG AGC	RGDLGLS
P2	26	TGC GAT TGC CGC GGC GAT TGC TTT TGC	CDCRGDCFC
P4	50	AAC GAT GTG CGC AGC GCG AAC	NDVRSAN
P5	50	AAC GAT GTG CGC GCG GTG AGC	NDVRAVS
A1	19	ATG CCA TTA GGA GCG GCA GGC	MPLGAAG
A2	19	AAC TAC TCC AGA GGA GTG GAC	NYSRGVD
A6	19	AAC GAG GCG CGG GTC CGG GAG	NEARVRE

[a] *Forward oligonucleotides encoding the peptides.*

Supplementary Table S2: Peptide insertion sites in the different AAV serotypes screened. Table adapted from Boerner *et al.*, 2020.[19]

Capsid	Insertion site [a]
AAV1	(i) D590_P591<br>(ii) S588_T589
AAV2	(i) R588_Q589<br>(ii) N587_R588
AAV3	(i) S586_S587<br>(ii) N588_T589
AAV4	(i) S584_N585<br>(ii) S586_N587
AAV5	(i) S575_S576<br>(ii) T577_T578
AAV6	(i) D590_P591<br>(ii) S588_T589
AAV7	N589_T590
AAV8	N590_T591
AAV9	Q588_A589
AAVrh.10	N590_A591
AAVpo1	(i) N567_S568<br>(ii) N569_T570
AAV12	(i) N592_A593<br>(ii) T594_T595

[a] *The peptide insertion sites are represented with flanking residues in the one-letter amino acids code. Depending on the serotype, more than one insertion site has been targeted.*

Supplementary Figures

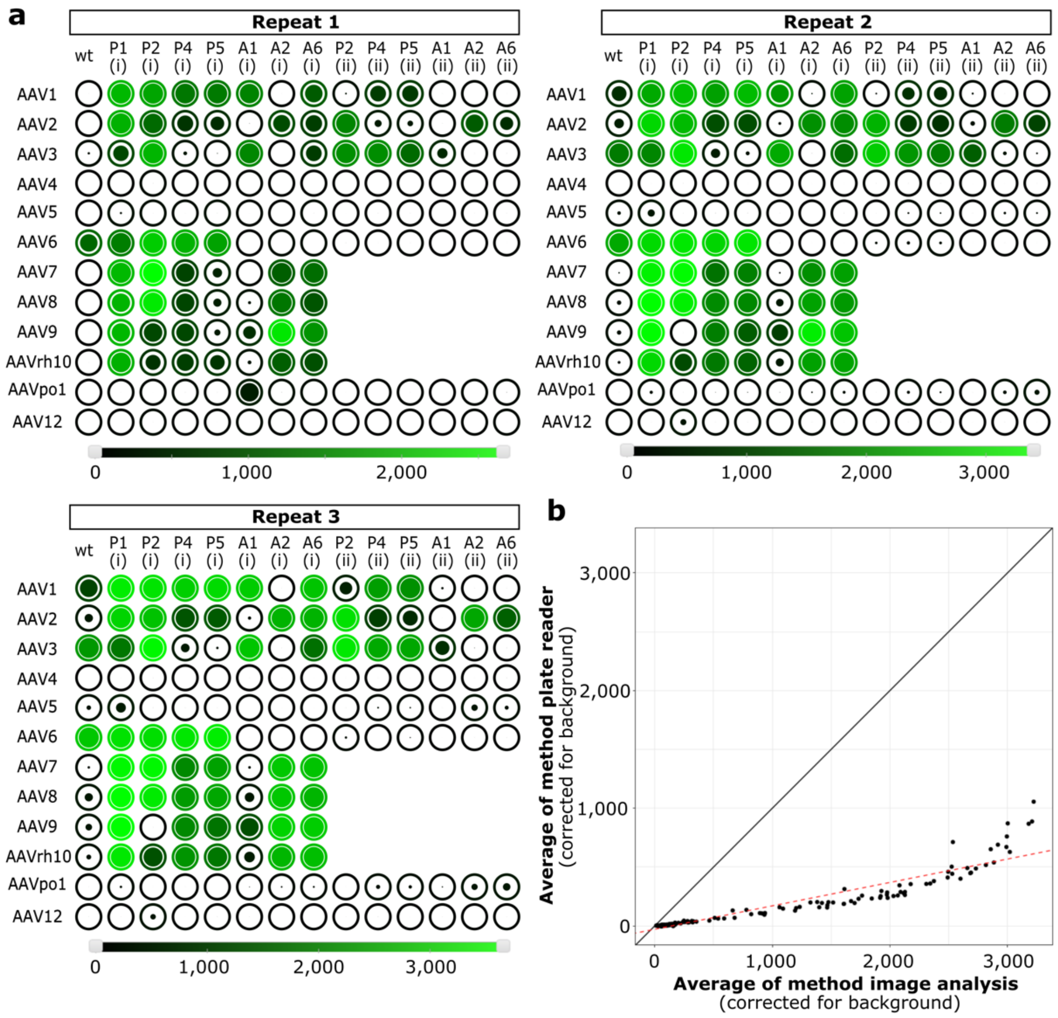

Supplementary Figure S1: Comparison of fluorescence measurements across independent experimental replicates (a) and using two different readout methods (b).

(a) Bubble charts representing transduction efficiencies (circle filling; in %) and absolute expression levels (color scalebar; mean gray value of all cells in the analyzed images) of all screened AAV-YFP variants in directly converted iNSCs. Each bubble chart is based on the quantification of immunofluorescent images resulting from one single screening run. The experiment was repeated in three individual runs (Repeats 1-3; all different from the independent experiment shown in Fig. 1c). The reliability of this readout method was statistically assessed by calculating intraclass correlation coefficients in a two-way random effects model based on single units. Intraclass correlation coefficient for parameter transduction efficiency = 0.90 [lower bound: 0.86, upper bound: 0.93]; p = 2.3e-150. Intraclass correlation coefficient for parameter absolute expression levels = 0.79 [lower bound: 0.63, upper bound: 0.87]; p = 2.6e-111. **(b)** YFP fluorescence intensity was quantified after transduction of the AAV-YFP screening panel in directly converted iNSCs by different readout methods, *i.e.*,

fluorescence measurements using automated image acquisition and analysis pipelines (raw data depicted in panel a) or a plate reader. Intraclass correlation coefficient for fluorescence intensities quantified by plate reader = 0.76 [lower bound: 0.66, upper bound: 0.83]; p = 1.8e-86. The concordance between both methods was statistically assessed by calculating repeated measures agreement and is depicted using a line of identity plot. Concordance correlation coefficient = 0.262, 95 % C.I. [0.207, 0.316]. N = 3 independent experiments.

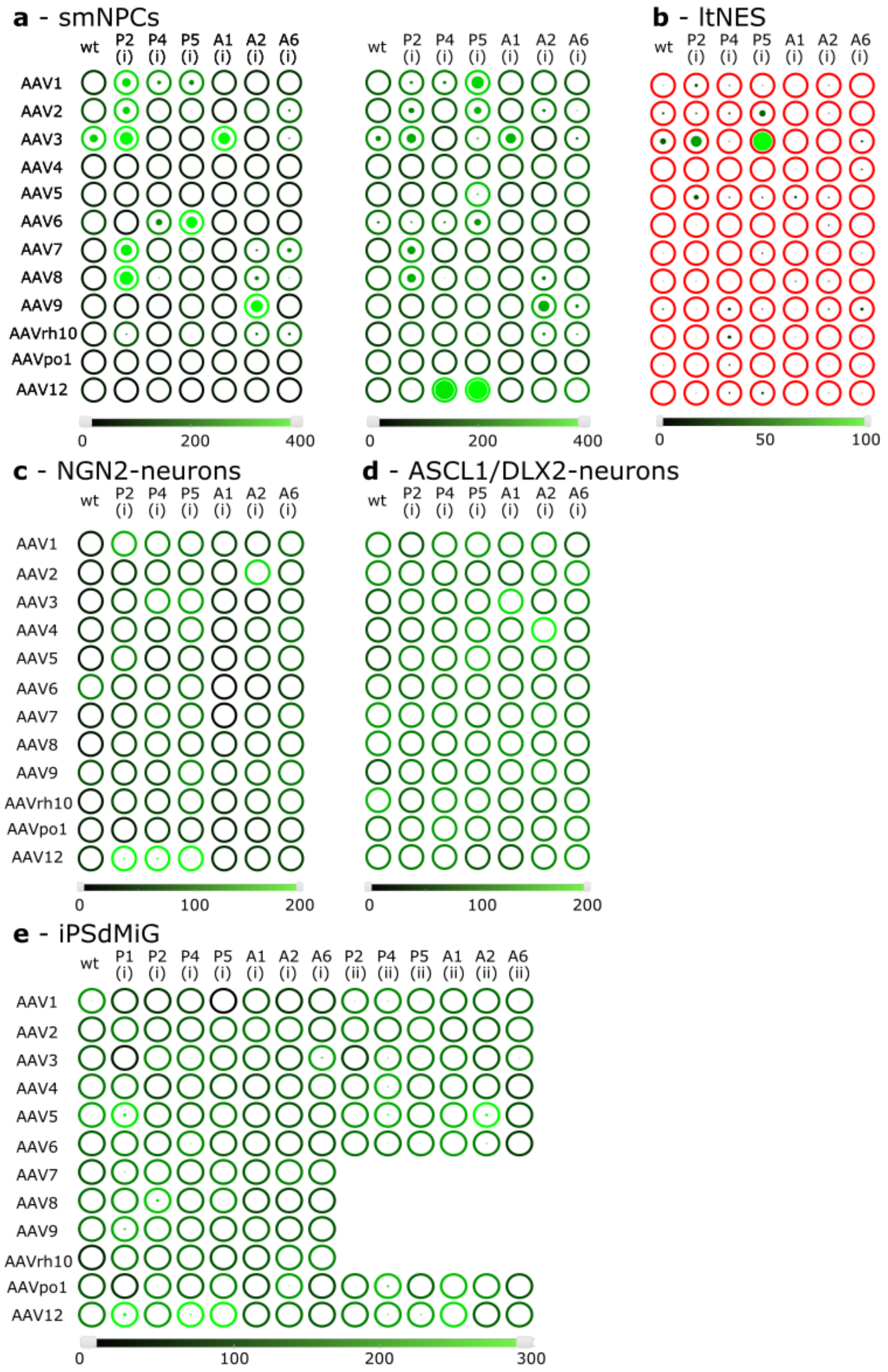

Supplementary Figure S2: Overview on the transducibility of human cell programming-derived CNS cell types.

(a - e) Bubble charts representing the transduction efficiencies of all screened AAV-YFP variants in smNPCs (a), undifferentiated ltNES (b), forward programmed NGN2- (c) and ASCL1/DLX2-neurons (d) as well as iPSdMiG (e). Panels a, c, d and e are based on the quantification of fluorescence intensities according to imaging data, whereas panel b is based on plate reader measurements. Plate reader-derived data are identified by red outlines. wt = wild-type. Inserted peptides (P1, P2, P4, P5, A1, A2, A6) and insertion sites (i, ii) are described in Supplementary Tables S1 and S2, respectively. Each bubble chart is based on the results of a single screening run.

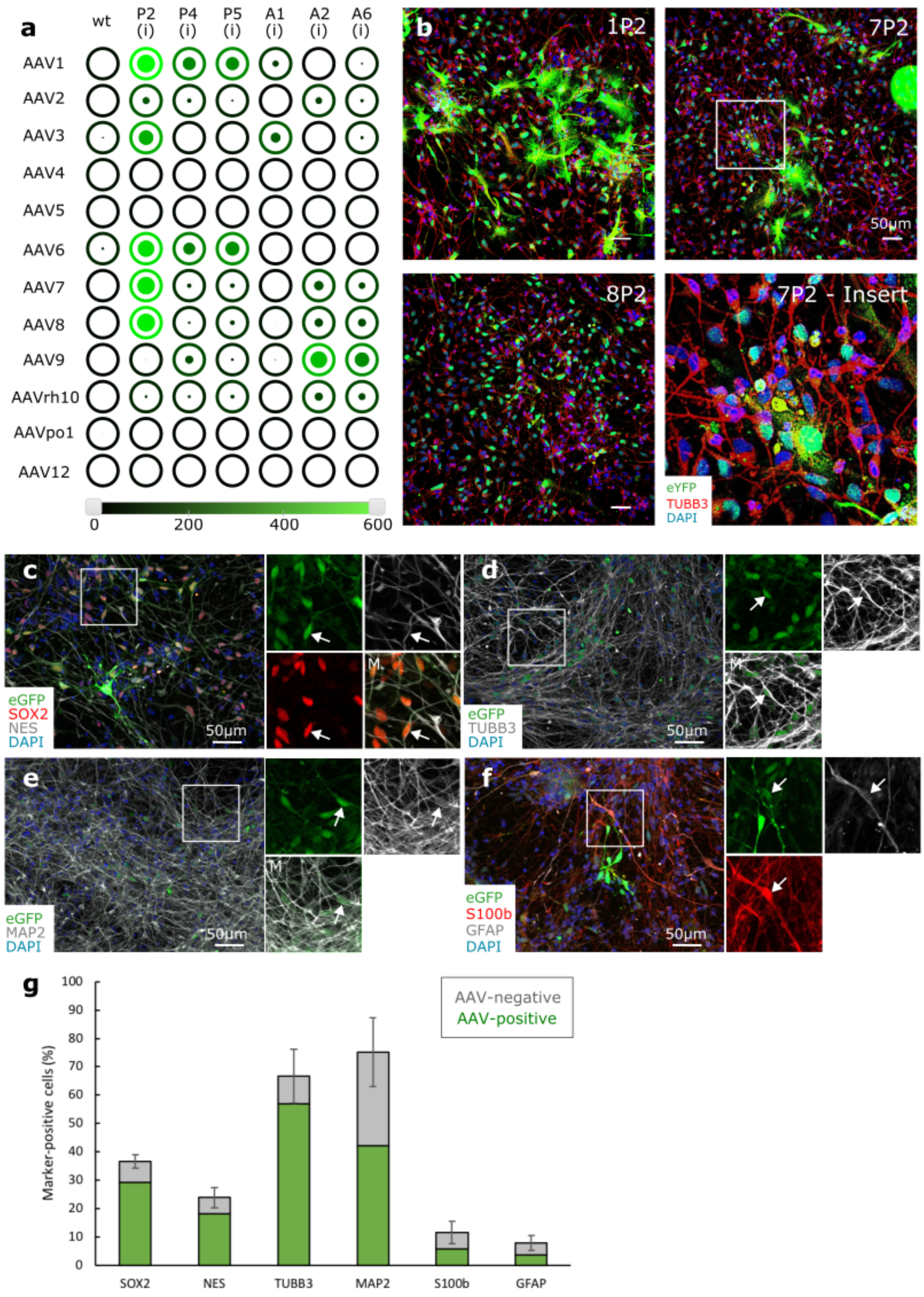

Supplementary Figure S3: Characterization of cell type-specific transduction in ltNES-derived neuroglial differentiation cultures.

(a) Bubble chart representing transduction efficiencies (cycle filling; in %) and absolute expression levels (color scalebar; mean gray value of all cells in the analyzed images) of all screened AAV-YFP

variants in ltNES-derived neuroglial differentiation cultures after immunofluorescence analysis with an antibody to TUBB3. The displayed bubble chart is based on the results of a single screening run, which represents an independent repetition of the experimental data provided in Figure 1 d. **(b)** Representative images illustrating the existence of TUBB3/YFP-double-positive cells in AAV screening plates, indicating the general capability of selected AAV variants to transduce neurons. **(c-f)** Representative pictures of ltNES-derived differentiation cultures after transduction with a pool of three GFP-encoding AAVs (AAV1P2, 7P2 and 8P2). Immunofluorescence stainings were performed for markers of NSCs (SOX2, NES; c), differentially mature neurons (TUBB3, MAP2; d, e) and glial cells (S100b, GFAP; f). Arrows within the magnified areas (*i.e.*, white rectangles in the source picture) point toward examples of GFP-positive cells, which are also immunopositive for the assessed markers. Close-ups denoted with 'M' are merged overlays of the provided single channel pictures. **(g)** Quantification of immunofluorescence stainings shown in figure panels c-f. N = 5-6 images per marker.

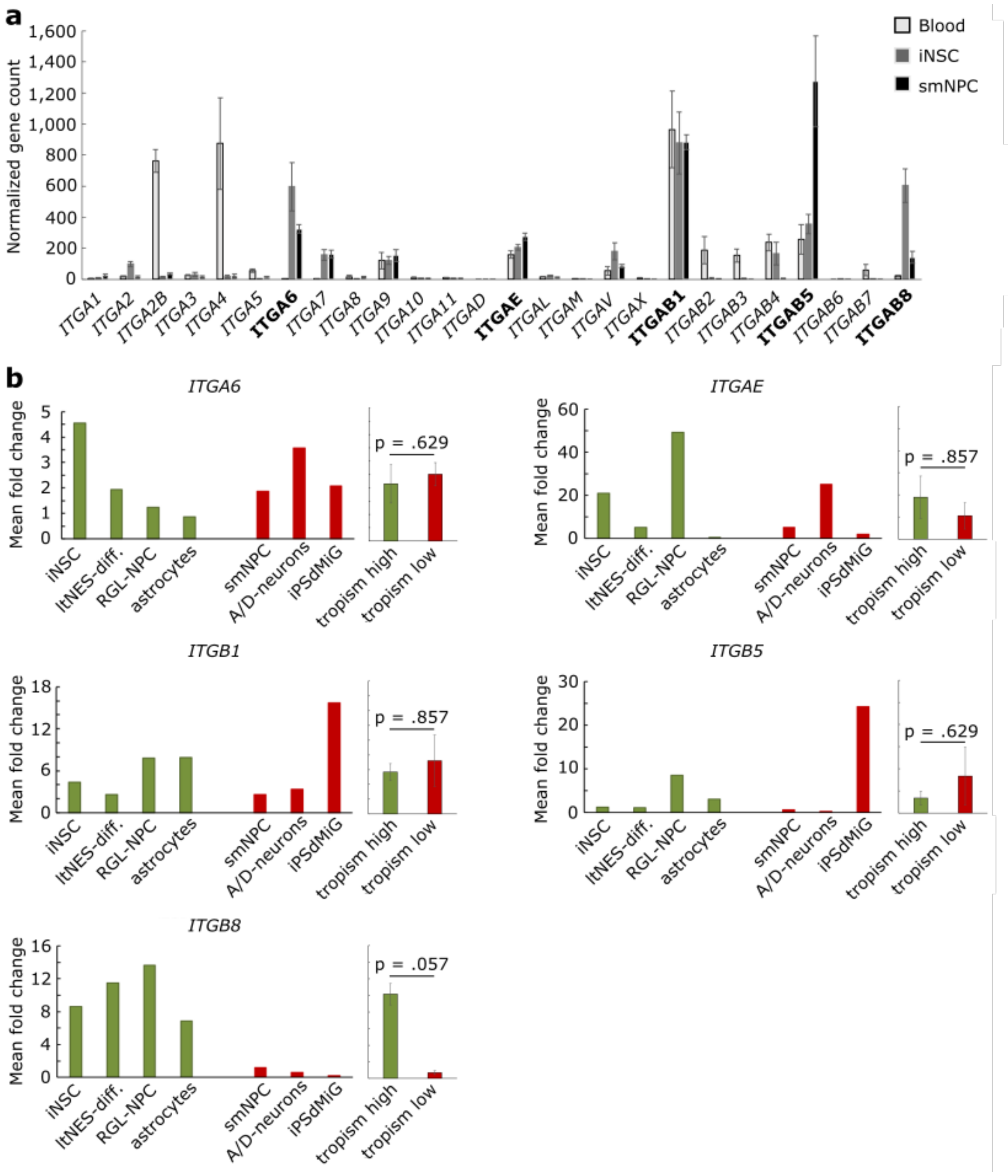

Supplementary Figure S4: Integrin expression in different human cell populations.

(a) Normalized gene counts of integrin family members in human blood cells, directly converted iNSCs and iPSC-derived smNPCs. RNA sequencing data used for this analysis have been published before.[20] Integrins printed in bold are the five family members with highest expression levels in iNSCs. Bar graphs represent means ± standard error with N = 3-9 independent samples per group. **(b)** qPCR-based expression profiling of the five integrins with highest gene counts in iNSCs across different human cell programming-derived cell types. Left panels in each graph depict the individual data of all cell types studied here, whilst the panels on the right depict the means of all well or poorly transduced types (tropism high or low, respectively). Wilcoxon signed rank tests were performed on the pooled datasets. Bar graphs depict mean fold changes ± standard error, if applicable, normalized to commercial human fetal brain (18-19 weeks of gestation; Agilent Technologies, Santa Clara, USA). A/D-neurons = ASCL1/DLX2-induced neurons.

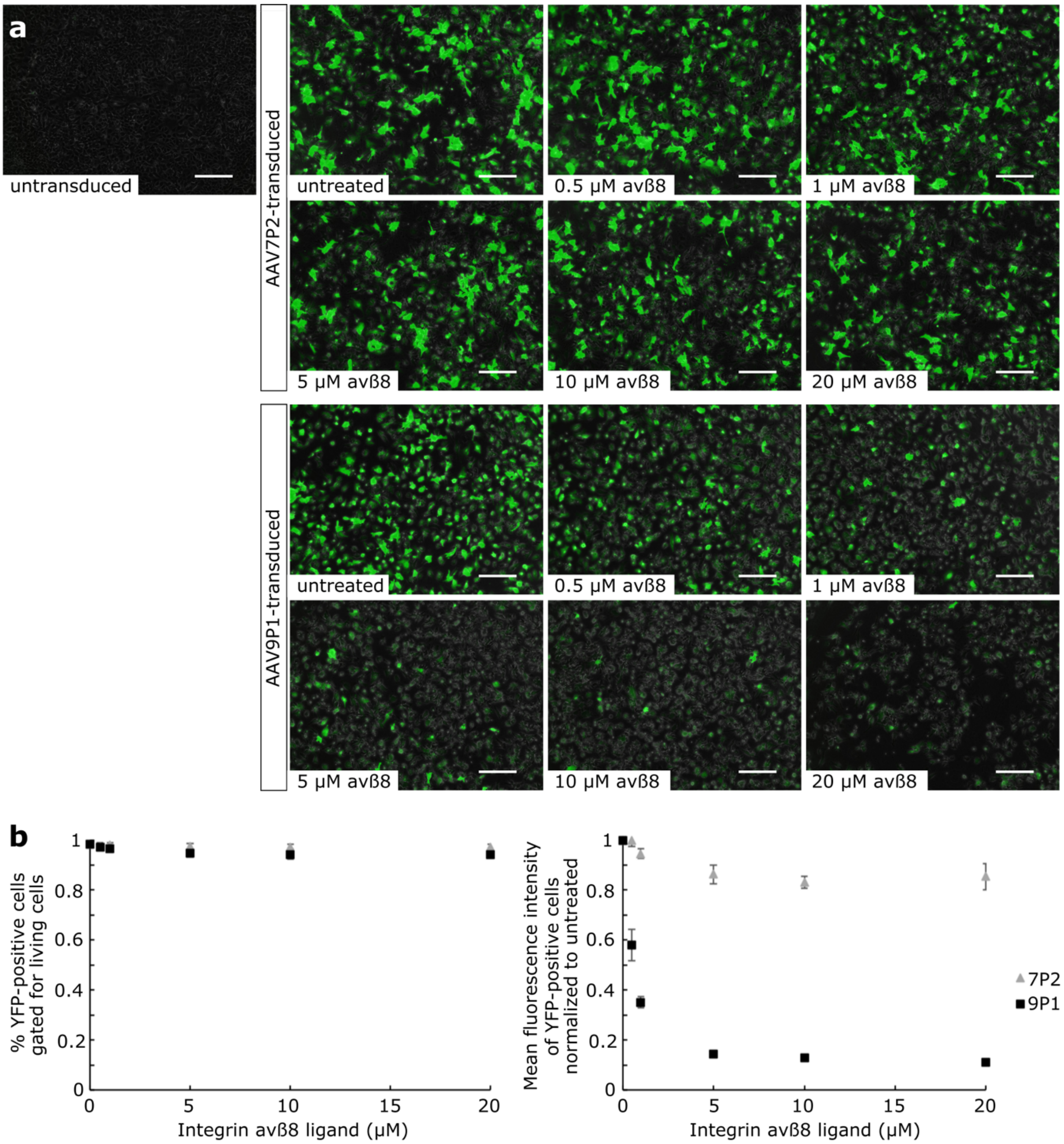

Supplementary Figure S5: AAV transduction efficiencies and YFP expression levels upon integrin αvβ8 inhibitor co-treatment.

(a) Representative overlays of live-cell phase contrast and fluorescence images, showing fluorescence levels in iNSCs 48 hours after AAV transduction and integrin αvβ8 ligand co-treatment. Pictures were captured prior to cell dissociation and subsequent flow cytometry analysis. N = 3 independent experiments. **(b)** Flow cytometry analysis revealing the overall percentages of YFP-positive cells (left) and the mean fluorescence intensities of AAV-transduced iNSCs (right) after 48 hours of incubation with concentrated AAV-YFP ± integrin αvβ8 ligand in various concentrations. Dot plots represent means ± standard error from N = 3 independent experiments. Correlation was statistically assessed using Kendall's τ: AAV7P2 transduction efficiency - Integrin αvβ8 inhibitor concentration: τ = -0.41, p = .023; AAV7P2 YFP intensity - Integrin αvβ8 inhibitor concentration: τ = -0.57, p = .002; AAV9P1

transduction efficiency - Integrin $\alpha v\beta 8$ inhibitor concentration: $\tau = -0.54$, $p = .003$; AAV9P1 YFP intensity - Integrin $\alpha v\beta 8$ concentration: $\tau = -0.92$, $p = 4.3e-07$.

Supplementary References

66. Michelfelder, S. *et al.* Successful expansion but not complete restriction of tropism of adeno-associated virus by in vivo biopanning of random virus display peptide libraries. *PLoS One* **4**, e5122 (2009).
